# Genome-Wide Analysis of *BBX* Gene Family in Three *Medicago* Species Provides Insights into Expression Patterns under Hormonal and Salt Stresses

**DOI:** 10.3390/ijms25115778

**Published:** 2024-05-26

**Authors:** Jiayin Wang, Zhuang Meng, Huan He, Pingping Du, Paul P. Dijkwel, Shandang Shi, Hongbin Li, Quanliang Xie

**Affiliations:** 1 Key Laboratory of Xinjiang Phytomedicine Resource and Utilization of Ministry of Education, Xinjiang Production and Construction Corps Key Laboratory of Oasis Town and Mountain-Basin System Ecology, College of Life Sciences, Shihezi University, Shihezi 832003, China; wjyinee@163.com (J.W.); zhuangmeng610@163.com (Z.M.); he_huan026@163.com (H.H.); dopingping@126.com (P.D.); shi_shandang@163.com (S.S.); 2 School of Natural Sciences, Massey University, Tennent Drive, Palmerston North 4474, New Zealand; p.dijkwel@massey.ac.nz

**Keywords:** BBX, alfalfa, transcription factors, gene expression, stress response

## Abstract

BBX protein is a class of zinc finger transcription factors that have B-box domains at the N-terminus, and some of these proteins contain a CCT domain at the C-terminus. It plays an important role in plant growth, development, and metabolism. However, the expression pattern of BBX genes in alfalfa under hormonal and salt stresses is still unclear. In this study, we identified a total of 125 *BBX* gene family members by the available *Medicago* reference genome in diploid alfalfa (*Medicago sativa* spp. *Caerulea*), a model plant (*M. truncatula*), and tetraploid alfalfa (*M*. *sativa*), and divided these members into five subfamilies. We found that the conserved motifs of *BBXs* of the same subfamily reveal similarities. We analyzed the collinearity relationship and duplication mode of these *BBX* genes and found that the expression pattern of *BBX* genes is specific in different tissues. Analysis of the available transcriptome data suggests that some members of the *BBX* gene family are involved in multiple abiotic stress responses, and the highly expressed genes are often clustered together. Furthermore, we identified different expression patterns of some *BBX* genes under salt, ethylene, salt and ethylene, salicylic acid, and salt and salicylic acid treatments, verified by qRT-PCR, and analyzed the subcellular localization of *MsBBX2*, *MsBBX17*, and *MsBBX32* using transient expression in tobacco. The results showed that *BBX* genes were localized in the nucleus. This study systematically analyzed the *BBX* gene family in *Medicago* plants, which provides a basis for the study of *BBX* gene family tolerance to abiotic stresses.

## 1. Introduction

Eukaryotic organisms always combine with multiple protein factors to co-regulate the transcription process. Zinc finger transcription factor is one of the largest families of transcription factors in eukaryotes, which can be divided into several subfamilies according to the structure and function of its members. B-box (BBX) is one of the subfamilies, and it is an important factor in the regulation of plant growth and development. BBX proteins have one or two B-box domains at the N-terminus of the protein, with the domain containing a zinc-binding motif [1], and the C-terminus is often accompanied by a CCT domain (for CONSTANS, CO-LIKE, TOC1 domain) [2,3]. During evolution, segmental duplication and gene deletion have led to variability among the *BBX* gene family [4,5,6]; this enriches the family’s genetic function.

In recent years, the *BBX* gene family has attracted much attention due to its diverse functions. Studies have been carried out on the model plants *Arabidopsis* [7], tomato [8], rice [9], cotton [10], grape [11], and other species. *BBX* is involved in plant photomorphogenesis [12], stomatal opening movement [13], floral development [14], and various abiotic stress responses such as drought, heat, and salt [8,15,16]. The B-box conserved domain in BBX proteins interacts with other proteins to perform photomorphogenic building functions [7]. In *Arabidopsis*, *AtBBX25* regulates photomorphogenesis in seedlings by interacting with ELONGATED HYPOCOTYL5 (HY5), and co-regulates the plant de-etiolation process and shade avoidance response with its homologue gene *AtBBX24* [17,18]. The *AtBBX24* gene not only can play a negative regulatory role in the blue light signaling pathway, but also binds to an H-protein promoter binding factor (HPPBF-1), which contains sequences encoding Myb DNA-binding motifs that are involved in plant defense stress responses [16,19,20,21]. In apple, *MdBBX37* inhibits HY5 expression and relieves growth inhibition of hypocotyls [22].

Abiotic stress is a major obstacle that inhibits plant growth and development, limits crop yield enhancement, and impairs plant photosynthesis [23]. Plants have developed various strategies to adapt to such stresses [24]. In *Arabidopsis*, the *AtBBX5* gene is regulated by ABA, mannitol, and salt induction. Overexpression of *AtBBX5* regulates ABA biosynthesis and metabolism-related gene transcript levels, which is a positive modulation of abiotic stress tolerance in plants through the ABA metabolic regulatory pathway [2]. The expression level of the *MdBBX10* gene was significantly increased by salt induction. Overexpression of the *MdBBX10* gene significantly enhanced tolerance to abiotic stress in *A. thaliana* [25,26]. *MdBBX37* enhanced cold tolerance in plants by mediating the jasmonic acid metabolic pathway [27]. In grapes, *VvBBX15a*, *VvBBX15b*, and *VvBBX22b* expression levels were dramatically up-regulated by ethylene (ETH), methyl jasmonate (MeJA), and salicylic acid (SA) [11]. In tomatoes, *SlBBXs* are induced and regulated by hormones such as auxin (IAA), gibberellin (GA), ETH, SA, and 6-BA [8]. Several studies have shown that the application of ethylene or salicylic acid can resist the damage caused by abiotic stresses to the plant itself. Ethylene participates in plant secondary metabolism, regulates photosynthesis and stomatal opening and closing, promotes chlorophyll degradation [28,29], and relieves the inhibition of seed germination by salt stress [30]. Salicylic acid can affect plant growth and development by participating in water metabolism, nutrient absorption, and the promotion of photosynthesis [31] to improve plant tolerance to abiotic stresses. However, *BBX* in *Medicago* plants in response to phytohormones and stress tolerance has rarely been reported.

Alfalfa (*Medicago sativa* L.) is one of the most widely grown perennial legume plants, abundant in vitamins, proteins, and minerals, low in calories, and high in productivity, and has excellent grass quality. It is an economic crop that can potentially improve global food security [32]. Improving its resistance is extremely critical to global ecosystems and sustainable agriculture. Perennial herbaceous autotetraploid alfalfa (*M*. *sativa*, 2n = 4x = 32) has a complex genome that is difficult to assemble [33]. However, genomic data on diploid alfalfa (*Medicago sativa* spp. *Caerulea*, 2n = 2x = 16) have been released in recent years. Diploid alfalfa, the progenitor of autotetraploid alfalfa, is more complete and could be an essential addition to the tetraploid alfalfa genome. Diploid alfalfa diverged from *M. truncatula* (2n = 16) about 5.2 million years ago, and chromosomal mutations have occurred during the process of differentiation [34].

In order to explore the function of the *BBX* gene family in *Medicago* plants more systematically and adequately, the genomic data of diploid alfalfa [34], *M. truncatula* [35], and *M. sativa* [33] were selected as the database of this study. We performed a genome-wide characterization of the *BBX* family in *Medicago* plants. Further, we analyzed the evolutionary relationship, gene structure, chromosomal localization and collinearity of the family members. Based on the published transcriptome data, the gene expression patterns in different tissues and under various abiotic stress treatments in alfalfa were also analyzed. Some BBX *genes* were selected for qRT-PCR expression analysis and subcellular localization identification to clarify the specific location of the family member exercising functions in the alfalfa. The results of this study provide clues for further exploring the specific functions of *BBX* in *Medicago* plants and provide candidate genes and the theoretical basis for molecular breeding efforts in highly stress-resistant alfalfa.

## 2. Results

### 2.1. Identification of BBX Genes

To deeply investigate the function of *BBX* genes, we downloaded the genome data files of three types of *Medicago* plants (*M. sativa* spp. *Caerulea*, *M. truncatula*, and *M. sativa*) from public databases. We identified a total of 125 members of the *BBX* family including 21 *MaBBX* genes, 25 *MtBBX* genes, and 79 *MsBBX* genes. All of these members have typical B-box conserved domains in the *BBX* family, which were named according to the order of chromosomal localization. We counted the detailed information of the 125 *BBX* genes (Table S1), including gene ID, gene name, chromosomal distribution, isoelectric point (pI), molecular weight (MW), number of amino acids (aa), instability index (II), aliphatic index (AI), grand average of hydrophobicity (GRAVY), and predicted position of subcellular localization.

The results showed that the number of amino acid sequences and MW of these BBX proteins spanned a wide range (Table S1). It ranged from a minimum of 79 aa and 9.04 kDa (MsBBX63) to a maximum of 521 aa and 57.572 kDa (MtBBX7). Only MsBBX63 and MtBBX10 protein-coding sequences were below 100 aa, while the only member above 500 aa was MtBBX7. Subsequently, the isoelectric points (pIs) of these BBXs were studied (Table S1). There are 100 BBX members with a pI lower than 7, ranging from 4.28 (MaBBX18) to 6.93 (MsBBX73). It is considered to be an acidic protein. The other 25 BBXs have pIs higher than 7, ranging from 7.02 to 9.74 (MsBBX63, MsBBX40, MsBBX53, MsBBX29, MsBBX30, MsBBX36, MaBBX10, MtBBX12, MsBBX43, MsBBX47, MaBBX14, MsBBX55, MsBBX57, MsBBX59, MsBBX61, MtBBX20, MsBBX42, MsBBX45, MsBBX49, MsBBX50, MsBBX51, MtBBX15, MaBBX16, MtBBX16, and MsBBX7), and are essentially basic proteins. Finally, the BBX protein members were analyzed by the instability index (Table S1). The results showed that only MsBBX63 and MtBBX10 were judged as stable proteins with instability index values less than 40. The other 123 BBXs were unstable proteins. The aliphatic index was distributed between 51.36 (MsBBX27) and 87.72 (MsBBX63), indicating that the thermal stability of these proteins was varied. All these BBX proteins had negative GRAVY values, showing that these proteins are hydrophilic. Based on Cell-PLoc server prediction, 125 BBX proteins were localized in the nucleus (Table S1).

### 2.2. Phylogenetic Analysis of BBX Proteins

**Figure 1:**
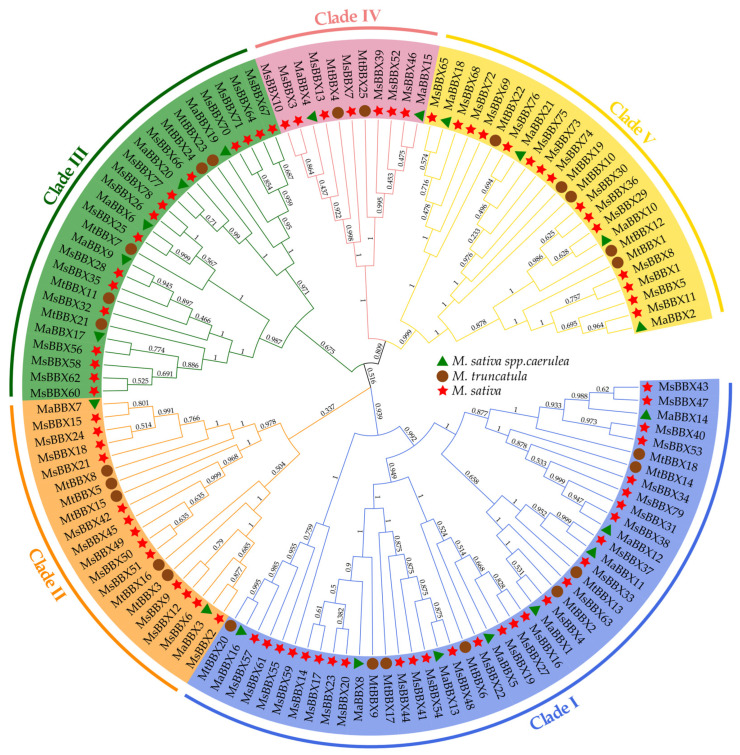
Phylogenetic relationships of BBXs retrieved in the *M*. *sativa* spp. *Caerulea*, *M. truncatula,* and *M*. *sativa*. The tree was constructed by Clustal W and MEGA X using the neighbor-joining method with 1000 bootstrap repetitions. The *BBXs* were classified and displayed in different groups by uniquely colored outer rings. The green triangle represents the *BBXs* in *M. sativa* spp. *Caerulea*; the yellow–brown circle represents the *BBXs* in *M. truncatula*; the red five-pointed star represents the BBXs in *M. sativa*. The number on the node in the phylogenetic tree represents the percentage of trustworthiness of the branch in the bootstrap validation.

To determine the evolutionary relationship of *BBX* family genes, we integrated a total of 125 full-length amino acid sequences of BBX proteins and constructed a phylogenetic tree based on 1000 bootstrap replications with the neighbor-joining method in the MEGA 10.2.6 software (Figure 1). According to our results, *BBX* members were clustered into five subfamilies based on evolutionary relationships. Among them, subfamily I contained 44 *BBXs*, forming the largest cluster. Subclade IV comprised the lowest number of *BBXs*, only 11. The remaining subclades II, III, and V contain 20, 26, and 24 members of *BBX*, respectively. The amino acid sequences of each subfamily share similarities and each BBX contains a B-box domain. Among them, most members of subfamilies I, III and V contain two B-box domains. Almost all subclade III, IV, and V members possess CCT domains. In particular, both clades II and IV contain only two *MaBBXs*, which are more clustered in the other subclades. Subclades II, III, and V contained five *MtBBXs*, suggesting a more average evolutionary distribution of *MtBBXs*. According to the evolutionary clustering relationship, it can be seen that diploid alfalfa *MaBBXs* evolved closely with tetraploid alfalfa *MsBBXs*.

### 2.3. Conserved Motif, Domains, and Gene Structure Analysis of BBX Genes

We analyzed the motif, domain, and exon–intron structural composition of 125 *BBX* sequences (Figure S1). Based on phylogenetic relationships, the 125 *BBX* family members were grouped into five subfamilies (Figure S1A). A total of 10 motifs were identified by the MEME algorithm (Figure S1B). The motif composition of each subfamily was different, and most members of the same subfamily showed similar motif structures. The complete B-box 1 domain usually consists of motif 1 and motif 5, the B-box 2 domain consists of motif 3, and the CCT domain consists of motif 2. In subclade I, *BBX* genes mostly contain B-box 1 and B-box 2 domains. Most members of clade II contain only the B-box 1 domain. Subclade III basically contains B-box 1, B-box 2, and CCT domains. Subclade IV contains B-box 1 and CCT domains. Clade V contains two B-box and CCT domains (Figure S1C). All BBXs contained the B-box 1 domain, and 84 BBX members contained the B-box 2 domain, accounting for 67.2% of all BBX proteins; 57 BBXs contained CCT structural domains, accounting for 45.6%. In particular, in clade V, five members (MsBBX65, MsBBX68, MsBBX69, MsBBX72, MtBBX22) contained two identical B-box 1 structural domains. The large number of duplications of this domain during evolution suggests that this domain has special significance in the regulation of certain physiological functions.

Based on the exon analysis, the number of exons in the *BBX* genes was more diverse (Figure S1D), ranging from one to seven. Among the *BBX* genes that clustered more similarly, their exon–intron structures were similar. Among these genes, 9, 37, 49, 23, and 5 *BBX* members contained 1–5 exon structures, accounting for 7.2%, 29.6%, 39.2%, 18.4%, and 4% of all *BBXs*, respectively. Only *MaBBX16* contained six exons, accounting for 0.8%. *MtBBX7* contained seven exons, which was the gene with the most exons in the *BBX* gene family, accounting for 0.8%. Overall, *BBX* members in the same subfamily show similar evolutionary patterns, and *BBX* genes are usually conserved in evolution.

### 2.4. Chromosomal Localization Analysis of BBX

**Figure 2:**
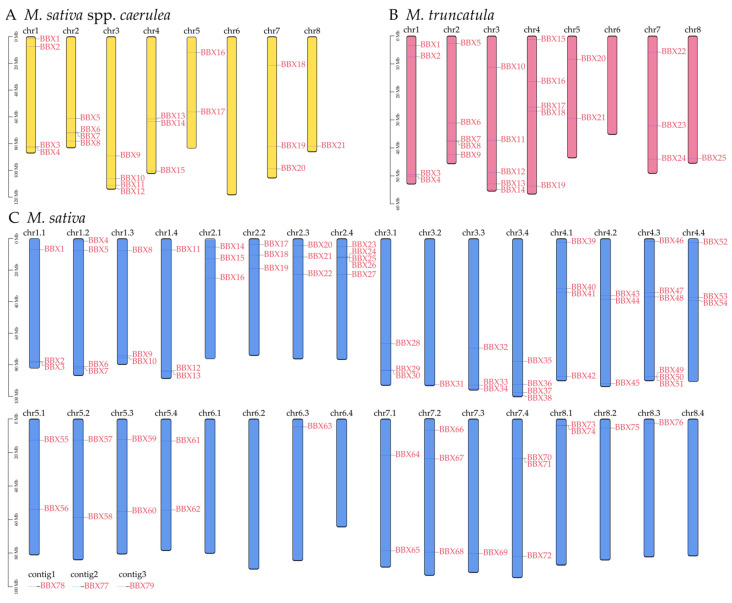
Chromosomal localization analysis of *BBX* in *M. sativa* spp. *Caerulea*, *M*. *truncatula,* and *M*. *sativa*. (**A**) The orange bar indicates the *M. sativa* spp. *Caerulea* chromosomes; (**B**) the pink bar indicates the *M. truncatula* chromosomes; (**C**) the blue bar indicates the *M. sativa* chromosomes. The gene on each chromosome is highlighted in red. The scale bar on the left indicates the chromosome lengths (Mb).

To understand the position of *BBX* genes, we analyzed the chromosomal localization of the *BBX* genes (Figure 2). The *BBX* genes were unevenly scattered on each chromosome, and the number of *BBX* distributions across each chromosome was not directly related to the length of the chromosome. In diploid alfalfa (*M. sativa* spp. *Caerulea*), *MaBBXs* were distributed over all chromosomes except chr6 (Figure 2A). Chr1, chr2, and chr3 all had four *MaBBX* genes. Chr4 and chr7 each had three *MaBBX* genes distributed. Chr5 had two *MaBBX* genes distributed. Chr8 had only *MaBBX21* distribution. Similarly, within the genome of *M. truncatula*, 25 *MtBBXs* were unevenly distributed over the other seven chromosomes, except on chromosome 6 (Figure 2B). Among them, five *MtBBX* genes were found on chr2, chr3, and chr4. Four, two, three, and one *MtBBX* genes were found on chr1, chr5, chr7, and chr8, respectively.

In *M. sativa*, 76 *MsBBX* genes were distributed over chromosomes, and three genes, *MsBBX77*, *MsBBX78*, and *MsBBX79*, were present in contig1, contig2, and contig3 (Figure 2C). Six genes (*MsBBX46-51*) were distributed over chr4.3, which is the chromosome with the highest distribution of *MsBBXs*. Five genes (*MsBBX23–27*) were localized on chr2.4. Four *MsBBXs* were localized on chr1.2, chr3.4, and chr4.1. There were 12 chromosomes that had three *MsBBXs* localized; they were chr1.1, chr1.3, chr1.4, chr2.1, chr2.2, chr2.3, chr3.1, chr3.3, chr4.2, chr4.4, chr7.2, and chr7.4. Two *MsBBX* genes were localized on chr5.1–5.4, chr7.1, and chr8.1, while on five chromosomes, only one *MsBBX* was localized; they were chr3.2 (*MsBBX31*), chr6.3 (*MsBBX63*), chr7.3 (*MsBBX69*), chr8.2 (*MsBBX75*), and chr8.3 (*MsBBX76*). Four of the chromosomes also had no *MsBBX* distribution: chr6.1, chr6.2, chr6.4, and chr8.4. In particular, on chr6.1 to 6.4, only *MsBBX63* was present, which may be an independently evolved gene that has undergone unique selective pressure compared to the other *MsBBXs*.

### 2.5. Analysis of the Synteny and Gene Duplication Event of the BBXs

**Figure 3:**
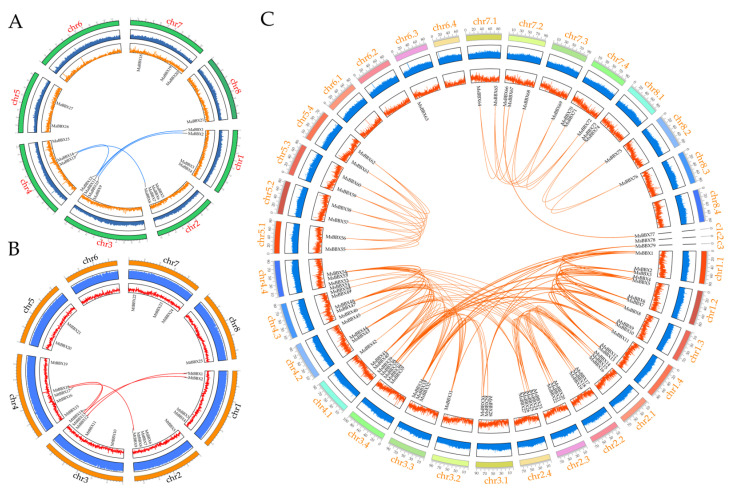
The synteny analysis of *BBXs* in the genomes of *M. sativa* spp. *Caerulea*, *M. truncatula,* and *M. sativa.* (**A**) The collinear relationship of *BBX* genes in *M. sativa* spp. *Caerulea*; (**B**) the collinear relationship of *BBX* genes in *M. truncatula*; (**C**) the collinear relationship of *BBX* genes in *M. sativa*. The *BBX* collinear gene pairs are represented by different colored lines. The outermost circle shows the chromosome number. The information represented by each circle in the figure from inside out is gene density, GC content, chromosome length scale, and chromosome name in order.

We calculated the collinearity, GC content, and gene density within the genomes of the three *Medicago* plants and mapped the Circos plots (Figure 3). A total of 116 pairs of *BBX* genes were found to have a collinear relationship from the genome database (Table S2). Among them, there were five homologous gene pairs in *M. sativa* spp. *Caerulea* and *M. truncatula*. Next, we calculated the non-synonymous (Ka) and synonymous substitution (Ks) and the ratio of Ka/Ks for all gene pairs to analyze the selective pressure on the evolution of the family. The results showed that Ka/Ks values ranged from 0.023 to 1.172, indicating that most members of the *BBX* family experienced strong purifying selection and had different evolutionary rates. Only two gene pairs in *M. sativa* (*MsBBX31* and *MsBBX38*, *MsBBX39* and *MsBBX52*) had Ka/Ks ratios greater than one, meaning that these two pairs of homologous genes have experienced strong positive selection and they were rapidly evolving genes. The evolution of these two pairs of genes has extremely significant biological implications in this gene family.

**Figure 4:**
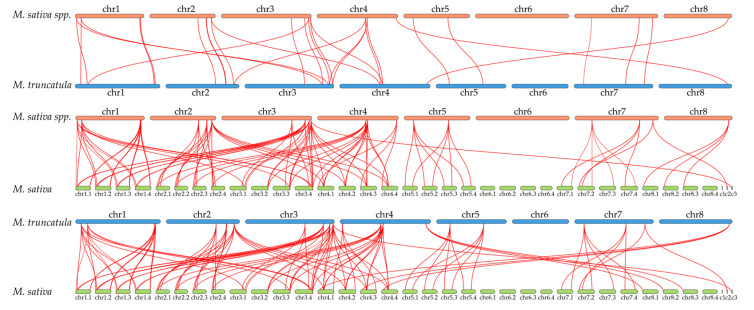
The synteny analysis of *BBXs* in the genomes between the *M. sativa* spp. *Caerulea*, *M*. *truncatula,* and *M*. *sativa*. The red lines represent homologous gene pairs between them.

We analyzed these 125 *BBX* family genes for duplication events (Table S3). The results showed that there were no tandem duplication events of *M. sativa* spp. *Caerulea* and *M. truncatula*, whereas eight members of each were duplicated by WGD or segmental duplications to expand the family members; 13 and 16 genes, respectively, were duplicated by dispersed duplications. In particular, only *MtBBX15* is a singleton gene. Dispersed duplications are the main evolutionary pattern for *MaBBX* and *MtBBX* genes. However, analysis of *BBX* family genes of *M. sativa* revealed a more diverse pattern of gene amplification. Two genes (*MsBBX63*, *MsBBX78*) were dispersed duplications (2.53%). Three genes (*MsBBX50*, *MsBBX51*, *MsBBX71*) were tandem duplication events (3.80%). Four genes (*MsBBX25*, *MsBBX26*, *MsBBX30*, *MsBBX74*) were proximal distributions (5.06%). The remaining 70 genes were WGD or segmental duplications, accounting for 88.61%. These findings show that the main cause of gene expansion in alfalfa is WGD or segmental duplications. In addition, we also performed collinearity analysis on *BBX* members among three *Medicago* plants separately (Figure 4). There were 29 collinearity gene pairs calculated between *MaBBX* and *MtBBX* genes; 101 gene pairs between *MaBBX* and *MsBBX* genes; and 105 gene pairs between *MtBBXs* and *MsBBXs*. We found that diploid alfalfa and *M. truncatula* genes can be mapped to 1–4 tetraploid alfalfa genes, suggesting that *Medicago* plant’s evolution may be a fourfold replication process.

### 2.6. Expression Patterns of BBX Genes in Different Tissues

**Figure 5:**
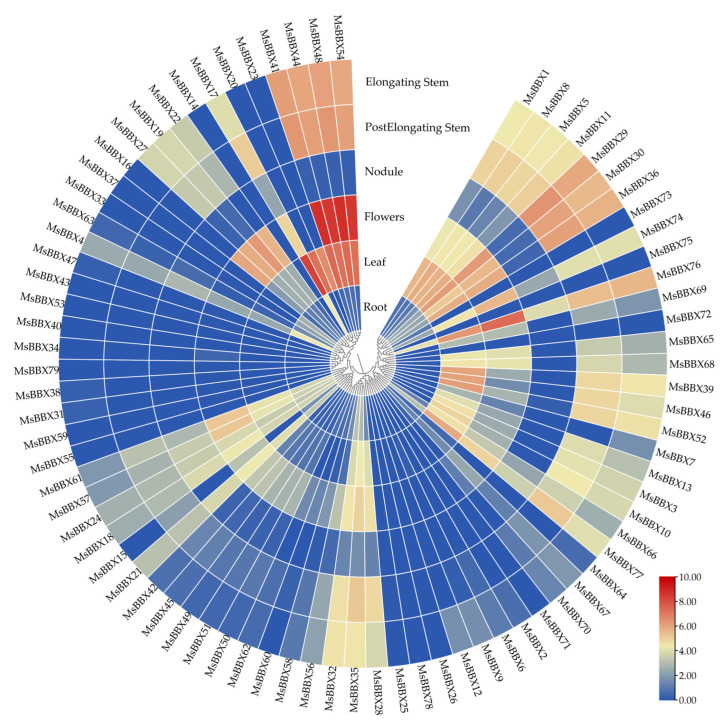
Expression profiles of *MsBBX* genes in different tissues of *M. sativa*. Heat maps reflect the transcripts per million (TPM) of *MsBBXs*. The color bar from blue to red indicates relative expression levels from lower to higher, respectively.

A heat map was constructed (Figure 5) using the public database of *M. sativa* in six different developmental tissues (roots, leaves, flowers, nitrogen-fixing root nodules, elongating stem internodes, and post-elongation stem internodes). The data results showed that the spatiotemporal expression patterns of these *MsBBX* genes were different in the various tissues.

In the flowering organs, most of the BBXs were expressed at a high level, especially *MsBBX41*, *MsBBX44*, *MsBBX48,* and *MsBBX54*. These *BBXs* may be involved in the developmental and metabolic regulatory processes of plant floral organs and play important roles. The levels of expression for *MsBBX41*, *MsBBX44*, and *MsBBX48* were also significantly higher than other *BBX* genes in leaves, elongating stem internodes, and post-elongating stem internodes. They clustered in the same evolutionary branch (Figure 1), which may possess similar functions in regulating plant development. In contrast, the gene expression was maintained at a lower threshold in roots and nitrogen-fixing root nodules. *MsBBX17*, the most highly expressed gene in roots, was approximately 1/21 of the most highly expressed gene in floral organs. The highest expressed gene in nodules, *MsBBX76*, was expressed lower than *MsBBX17* in roots. In addition, 11 genes (*MsBBX14*, *MsBBX26*, *MsBBX43*, *MsBBX53*, *MsBBX55*, *MsBBX59*, *MsBBX71*, *MsBBX72*, *MsBBX75*, *MsBBX78*, *MsBBX79*) were not expressed in any of these tissues. Significant gene expression differences in different tissues demonstrated that these family genes have a strong tissue-specific expression pattern, and they are involved in different alfalfa growth and developmental stages.

### 2.7. Expression Patterns of BBX Genes under Abiotic Stresses

**Figure 6:**
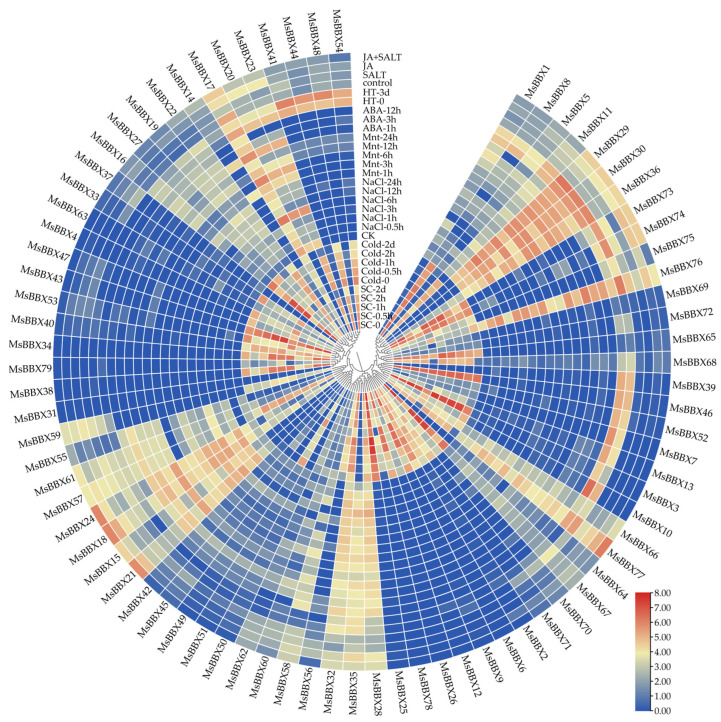
Expression profiles of *MsBBX* genes under different abiotic stresses and hormone treatments of *M. sativa*. Heat maps reflect the transcripts per million (TPM) of *MsBBXs*. The color bar from blue to red indicates relative expression levels from lower to higher, respectively.

The expression profiles of *MsBBXs* under various abiotic stresses were investigated using RNA-seq data. We summarized the heatmap analysis of the expression levels of 79 *MsBBX* genes under a variety of adversity stress conditions (cold, cold with salicylic acid, salt, drought, heat, abscisic acid, salt with jasmonic acid) using RNA-seq data that are publicly available online (Figure 6).

When alfalfa was subjected to low-temperature treatment, the expression levels of genes like *MsBBX35*, *MsBBX41,* and *MsBBX44* showed a tendency to decrease as the time increased. Most genes were also up-regulated at different times. For example, after 0.5 h of cold stress, the expression of *MsBBX3*, *MsBBX10,* and *MsBBX13* increased significantly compared with the control. Interestingly, alfalfa was watered using SA solution and then cold-treated (Figure 6). The expression of some *BBX* genes was higher than those that underwent cold treatment alone; it is possible that SA activated the expression of these genes. When the treatment was completed, alfalfa was placed in a culture chamber. After two days, the difference in gene expression was more significant in the cold-treated group with salicylic acid applied compared to the cold treatment alone. As in the case of *MsBBX46*, the expression content in cold treatment gradually decreased with time. After applying salicylic acid, the expression content of this gene was elevated. Removing all the stress factors for two days, its expression level far exceeded that of the cold-treated group at the same time. The results of these data indicate that salicylic acid can play an important role as a signaling molecule to activate plant resistance and defense mechanisms in the process of adversity stress in plants.

When treating alfalfa with salt, drought, and high-temperature conditions, different *BBX* genes showed differential expression patterns (Figure 6). Stimulation of alfalfa using NaCl significantly up-regulated some genes. For example, the expression of *MsBBX29* first gradually increased and then slowly decreased. However, after 24 h, the expression increased again. Some genes like *MsBBX17* and *MsBBX20* showed suppression in the initial stage and increased again with the extension of the treatment period. The expression modes of genes like *MsBBX24*, *MsBBX30,* and *MsBBX36* were increased by the induction of drought. At 6h, the expression of *MsBBX36* was about 3.6 times higher than the control. The level of expression of *MsBBX64* increased dramatically after high-temperature stress, which was as much as 24 times the original expression level. This gene responded positively to heat stress. Other genes, such as *MsBBX71*, *MsBBX73* and *MsBBX74*, also responded positively to high-temperature induction, and they were maintained at high levels.

Application of exogenous abscisic acid to alfalfa resulted in a significant increase in some genes like *MsBBX76* (Figure 6). After 1h of treatment, its expression level reached about 3.7 times that of ck, showing a high response pattern to exogenous ABA. Other *BBX* genes induced by ABA also showed different expression patterns, and these *BBXs* were involved in the ABA signaling pathway to regulate plant growth and metabolic processes. When alfalfa was treated with salt and jasmonic acid (Figure 6), most of the gene expression levels were increased compared with salt or jasmonic acid stimulation alone. For example, the expression level of *MsBBX18* under salt-induced conditions was half that under jasmonic acid-induced conditions but increased to 4-fold after synergistic induction. *MsBBX18* may be involved in the signaling pathways of salt and jasmonic acid. Meanwhile, this gene may also play an important role in the cross-regulatory pathway of these two abiotic stresses.

### 2.8. qRT-PCR Analysis of BBX Genes under Hormone and Salt Stresses

To confirm the regulation of salt tolerance among *MsBBX* gene family members by different hormones, alfalfa was used as the material; 150 mmol/L NaCl, 0.5 mmol/L ETH, and 150 mmol/L NaCl with 0.5 mmol/L ETH interaction treatments were set to analyze the relative expression of *MsBBX* genes. We selected 15 *BBX* genes that were responsive under a diverse range of abiotic stresses based on the results of transcriptome analysis (Figure 6). The expression was examined by quantitative real-time PCR (qRT-PCR) under NaCl, ETH, and NaCl with ETH treatments.

**Figure 7:**
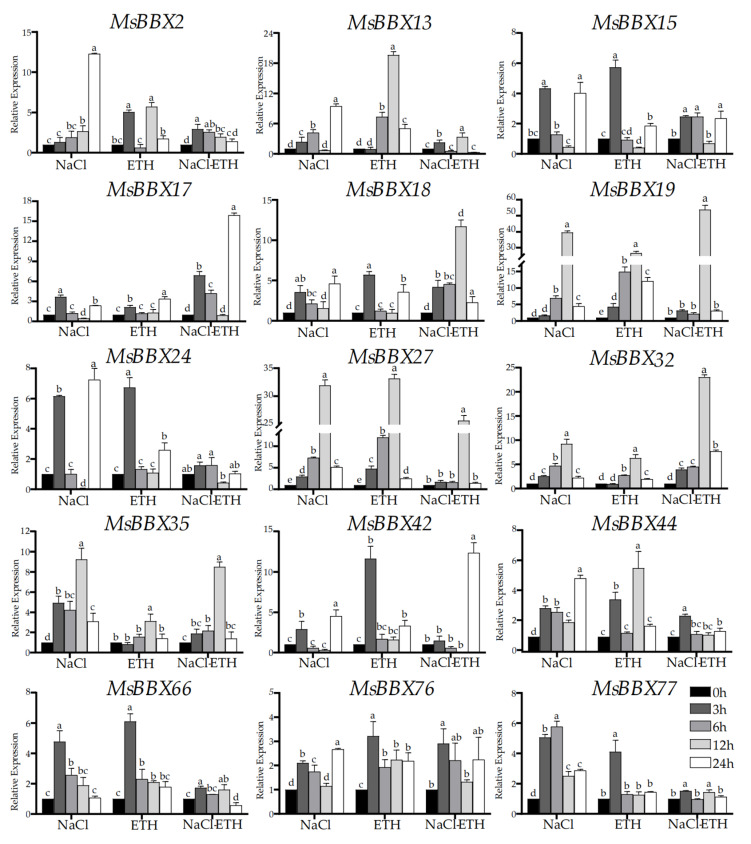
The expression profiles of 15 selected *MsBBX* genes in response to NaCl, ETH, and NaCl and ETH treatment in *M. sativa* by using qRT-PCR. Data are the average of three independent biological samples ± SD and vertical bars indicate standard deviation. Different letters indicate significant differences at *p* < 0.05, as determined by Duncan’s multiple range tests.

The results showed that *MsBBX* genes exhibited different expression patterns under varied treatment conditions as well as treatment times (Figure 7). In the NaCl treatment, the results showed that all gene expressions exhibited different degrees of up-regulation trends. In particular, *MsBBX19* and *MsBBX27* were most significantly up-regulated, followed by *MsBBX2*, *MsBBX13*, *MsBBX24* and *MsBBX35;* the expression of these four genes was also significantly increased after salt induction, indicating that these genes are involved in the process of the plant’s response to adversity. Under ETH treatment, *MsBBX* genes also showed different expression patterns; most of these genes were markedly increased after induction, suggesting that these genes are involved in the ethylene-regulated pathway. Under cross-stress treatment with NaCl and ETH, *MsBBX17*, *MsBBX18*, *MsBBX19*, *MsBBX32*, and *MsBBX42* showed higher relative expression levels at a certain time than when induced by NaCl or ETH alone. *MsBBX* genes synergistically regulate the adversity and hormone-induced pathways.

**Figure 8:**
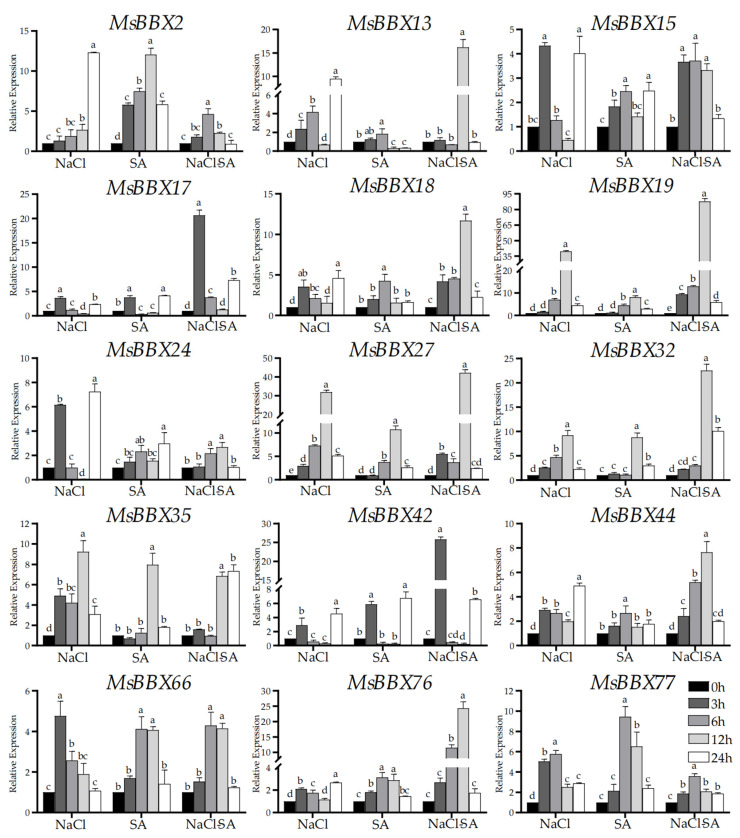
The expression profiles of 15 selected *MsBBX* genes in response to NaCl, SA, and NaCl and SA treatment in *M. sativa* by using qRT-PCR. Data are the average of three independent biological samples ± SD and vertical bars indicate standard deviation. Different letters indicate significant differences at *p* < 0.05, as determined by Duncan’s multiple range tests.

We used NaCl, SA, and the mutual treatment of both of them to detect the expression of *MsBBX* genes in alfalfa (Figure 8). Under the 0.5 mmol/L SA condition, the results showed that all *BBX* genes were up-regulated. *MsBBX2*, *MsBBX27*, and *MsBBX77* were the most notable genes. Some gene expression increased significantly with time, indicating that *MsBBX* genes were involved in the salicylic acid metabolic pathway. When alfalfa was treated cooperatively with 150 mmol/L of NaCl and 0.5 mmol/L of SA, most of the *BBX* genes showed higher levels of expression compared with those in the single stress treatments. For example, for *MsBBX19* and *MsBBX44* at 12 h, the expression was increased markedly higher than in single stress treatments. Some genes, such as *MsBBX2* and *MsBBX77*, had lower expression when they were subjected to cross-treatments. Induction of alfalfa using different approaches resulted in significant changes in gene expression levels for *BBX* members. *MsBBX* is involved in abiotic response processes in plants.

### 2.9. Subcellular Localization of BBX Genes

**Figure 9:**
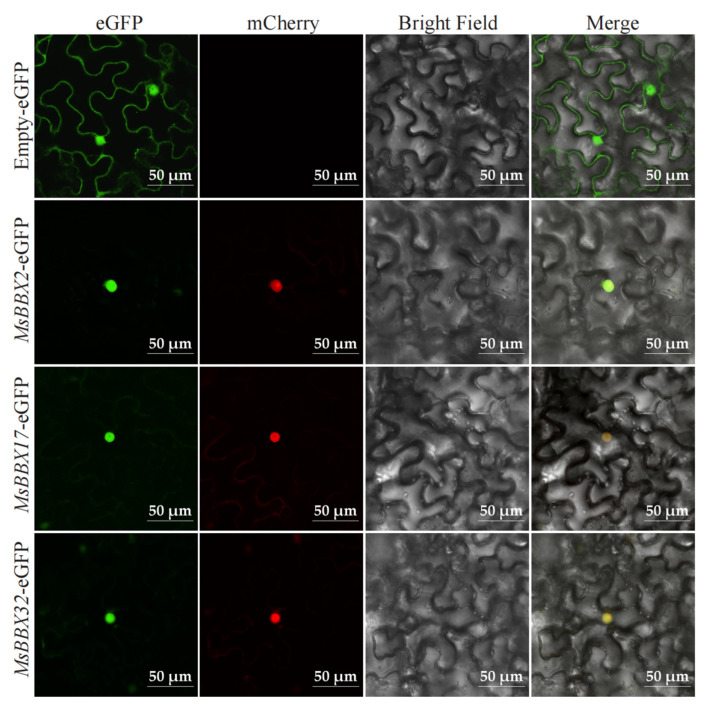
Subcellular localization of MsBBX2, MsBBX17, and MsBBX32. MsBBX:GFP fusion proteins were transiently expressed in *Nicotiana benthamiana* leaves, and their localization was determined using confocal microscopy. The nucleus was visualized with mCherry-labeled nuclear markers. Bar = 50 µm.

We predicted the subcellular localization of *BBX* on the Cell-PLoc website (Table S1). To further confirm the specific location where *BBX* exerts its function in the cells, we constructed recombinant vectors of 35S::eGFP fused with *MsBBX2*, *MsBBX17*, and *MsBBX32*. The empty-eGFP was the control. Fluorescence microscopy revealed that the GFP signals of all three fusion proteins were detected in the nucleus, consistent with Marker-mCherry (Figure 9).

## 3. Discussion

Transcription factors play key roles in plant life history, regulating plant growth, development, and responsiveness to abiotic stresses [36,37]. *BBX* is a branch of the zinc finger protein family of transcription factors involved in the regulation of plant flowering pathways [38], circadian rhythms [39], photomorphogenesis [40], and abiotic stress responses [6]. We systematically identified 21, 25, and 79 *BBX* members from *M. sativa* spp. *Caerulea*, *M. truncatula*, and *M. sativa* genomes, respectively (Table S1). Differences in the number of family members may be directly related to the chromosome number and genome complexity of different varieties [41], or interspecific differences may have occurred during evolution as a result of specific duplications or segmental deletions [11]. We listed some basic information about these *BBXs*, including gene ID, chromosomal localization, molecular weight, isoelectric point, and aliphatic index. These *BBXs* were divided into five branches (Clade I–V) based on the evolutionary pattern of the proteins, and it was found that *MaBBXs* and *MsBBXs* tended to cluster more closely (Figure 1).

We also analyzed the gene structure and conserved motifs of BBX (Figure S1). A total of 10 conserved motifs were identified, and the motif structures of each group member were relatively similar. Motif 1 was present in all *BBXs*. The *BBX* gene structures were rich and diverse (Figure S1D), with the number of exons ranging from one to seven. Nine of the *BBX* genes (*MsBBX42*, *MsBBX45*, *MsBBX49*, *MsBBX50*, *MsBBX51*, *MsBBX63*, *MsBBX5*, *MsBBX15*, *MsBBX16*) were without intronic structures, and they were almost all clustered together (Figure 1). The loss or gain of introns is an essential feature of species evolution [42,43].

The localization of these *BBX* genes on the chromosomes is very similar (Figure 2). For example, on chr1, the *BBX* genes are distributed at both ends of the chromosome; on chr5, two *BBXs* are distributed. Particularly, except for *MsBBX63*, the other 124 *BBXs* are scattered on chromosomes other than chr6, and the independent evolution of this gene may have enriched the function of the *BBX* gene family. Combined with the results of collinearity analysis (Figure 4), we found that one *MaBBX* or *MtBBX* gene could map to 1-4 genes in the tetraploid alfalfa genome, suggesting that there is a tetraploid duplication process in alfalfa. In *M. sativa* spp. *Caerulea* and *M. truncatula* (Table S3), dispersed duplications and WGD or segmental duplications were the main evolutionary modes. WGD or segmental duplications in *M. sativa* played a dominant role in the evolution of the *BBX* family (Table S3). It has been shown that WGD or segmental duplications are more favorable for the maintenance of gene function during amplification [41,44]. Exceptionally, only *MtBBX15* is a singleton gene, and may have some unique functions. In the process of evolution, gene duplication often undergoes some selective pressure as organisms adapt to the external environment [11]. Here, we calculated the homologous gene pairs present in the *BBX* genes of the three *Medicago* plants separately, and 106 pairs of collinear genes were calculated in *MsBBXs*, with five pairs of collinear genes in each of the other two plants (Table S2). By calculating the Ka/Ks ratio, it was found that only two gene pairs (*MsBBX31* and *MsBBX38*, and *MsBBX39* and *MsBBX52*) had Ka/Ks > 1, and the rest of the gene pairs had Ka/Ks values less than 1, suggesting that these *BBX* genes underwent purifying selection and that the proteins were relatively conserved [45,46]. Furthermore, *M. truncatula* diverged from *M. sativa* spp. *Caerulea* 5.2 million years ago, and tetraploid alfalfa evolved from diploid alfalfa [34]. It is likely that some chromosome splitting and fusion also occurred during the evolutionary process. The gene family identification in this paper shows that these species converged to the same trend in the family analysis, such as similar protein structure, chromosomal localization, and collinearity. And there are many different trends in the analysis results as well. For example, tetraploid alfalfa has a very large number of family members, almost four times as many as the other two species of alfalfa. Tetraploid alfalfa also has more complex and diverse collinear gene pairs, and so on. This is correlated with the complexity of the publicly available genomic data.

*MsBBX* genes showed specific expression patterns in different tissues (Figure 5), suggesting that these genes are involved in the developmental processes of multiple plant organs (Figure 5); 86.1% of *MsBBX* genes functioned in multiple processes of plant growth and development. Among them, most genes were expressed in elongating stem internodes, with the least in nodules. The expression patterns of these *BBXs* are high in flowering organs and leaves, and these high-level genes are often clustered together; for example, *MsBBX41*, *MsBBX44*, *MsBBX48*, and *MsBBX54* clustered in the same evolutionary branch. There are few studies on the pattern of *MsBBX* gene expression in alfalfa under a variety of adversity environments and hormones, whereas it has been studied in *Arabidopsis* [2], apple [27], grape [11], chrysanthemum [15], and other plants. Therefore, we downloaded the transcriptome data on alfalfa adversity stress and hormone treatment from the public data platform for analysis (Figure 6). The results showed that some of these *BBX* genes responded to multiple abiotic stresses under different treatments of cold, cold with salicylic acid, salt, drought, heat, abscisic acid, and salt with jasmonic acid. Among these highly expressed genes, some of them were located on the same chromosome (Figure 2), for example, *MsBBX28*, *MsBBX29*, and *MsBBX30;* the close gene distance may lead to similar gene functions. In particular, these genes showed significant differences in expression levels after being induced by two abiotic stress conditions simultaneously (Figure 6). The use of SA solution for watering alfalfa followed by cold treatment showed a different trend of increasing the expression levels of most of the *BBXs* compared with no application of SA, and these genes were synergistically regulated in the phytohormone and adversity stress pathways. By studying the expression mode of *BBX* genes, we found that some genes responded positively to various stress responses. For instance, *MsBBX18*, *MsBBX35,* and *MsBBX76* showed up-regulated tendencies when induced by salt, drought, heat, and hormones. These genes may be involved in multiple adversity and hormone-responsive metabolic regulatory pathways, which jointly regulate the growth and development process of alfalfa from multiple pathways.

We analyzed the expression of 15 *MsBBX* genes under different hormone and salt treatments by qRT-PCR (Figure 7 and Figure 8). All of these genes showed a tendency to be significantly induced by salt stress. If the exogenous hormones ethylene or salicylic acid were applied to alfalfa alone, similarly, these genes showed a differential tendency to be up-regulated, and these results are in agreement with the studies in other species [8,10,11]. However, when salt and ETH were used together to stress alfalfa, *MsBBX17*, *MsBBX*18, *MsBBX19*, *MsBBX32*, and *MsBBX42* showed higher expression levels than the treatments alone (Figure 7), while the expression levels of other *BBXs* were reduced compared to the control. These results suggest that *BBX* may be involved in the ethylene signaling pathway and may regulate its expression via the pathway under adverse environments. *MsBBX13*, *MsBBX17*, *MsBBX18*, *MsBBX19*, *MsBBX27*, *MsBBX32*, *MsBBX42*, *MsBBX44*, and *MsBBX76* showed up-regulated expression patterns when both salt and salicylic acid stress conditions were simultaneously applied to alfalfa (Figure 8). They were actively involved in salt and SA induction, and it is likely that these genes synergistically regulate the salt and SA signaling pathway. Finally, by subcellular localization experiments (Figure 9), we verified that *BBX* genes were located in the nucleus. This shows that *BBX* genes may function as transcription factors in the nucleus [8]. The increased expression level of *BBX* under abiotic conditions shows that they are likely to be extensively involved as transcription factors in the regulation of abiotic stress tolerance in plants, and are involved in the signaling pathways of a variety of phytohormones.

## 4. Materials and Methods

### 4.1. Genome-Wide Identification of MaBBXs, MtBBXs, and MsBBXs

For the identification of *MsBBX* genes, the *M. sativa* reference genome data were downloaded (https://figshare.com/, accessed on 6 August 2023) and the latest *M. truncatula* and *M. sativa* spp. *Caerulea* reference genome data were downloaded from the MODMS database (https://modms.lzu.edu.cn/, accessed on 6 August 2023) [47]. The *AtBBX* gene was retrieved as a query sequence. The 35 AtBBX protein sequences were used as query sequences to retrieve BBX protein sequences in *M. sativa*, *M. sativa* spp. *Caerulea,* and *M. truncatula* reference genomes, respectively, with e-values < 1 × 10^−5^. Duplicate sequences were removed and submitted to NCBI CD-Search (https://www.ncbi.nlm.nih.gov/cdd/, accessed on 6 August 2023) to predict structural domains with an e-value threshold of 0.01. Protein sequences that did not contain the B-box structural domain were removed. It was finally determined that *M. sativa* spp. *Caerulea* has 21 *MaBBX* members, *M. truncatula* has 25 *MtBBX* members, and *M. sativa* has 79 *MsBBX* members.

The molecular weight, theoretical isoelectric point, instability index, aliphatic index, and grand average of hydrophobicity of the BBX sequences were calculated using EXPASy (https://web.expasy.org/protparam/, accessed on 8 August 2023), which was used to predict and analyze the physicochemical properties of all BBX protein sequences in conjunction with information from the reference genome GFF file. The subcellular localization of BBX proteins was predicted and analyzed using the online tool Cell-PLoc (http://www.csbio.sjtu.edu.cn/bioinf/Cell-PLoc/, accessed on 8 August 2023).

### 4.2. Phylogenetic Analysis of BBX Genes

Phylogenetic analyses were performed using 21 MaBBX sequences in *M. sativa* spp. *Caerulea*, 25 MtBBX sequences in *M. truncatula*, and 79 MsBBX sequences in *M*. *sativa*, for a total of 125 full-length protein sequences. Multiple sequence alignment was conducted using Clustal W with default parameters. We constructed a phylogenetic tree based on the complete BBX protein sequences using the neighbor-joining method (N-J) as implemented in the MEGA 10.2.6 software with a bootstrap value of 1000 and all parameters kept at default values [48]. The phylogenetic tree was visualized and modified using iTOL (https://itol.embl.de/, accessed on 15 August 2023) [49].

### 4.3. Gene Structure, Conserved Domains, and Motif Composition of BBXs

The BBX conserved motifs were identified using the MEME online website (https://meme-suite.org/meme/tools/meme, accessed on 19 August 2023), with the maximum number of motifs set to 10 in the program settings and the remaining parameters kept at default values [50]. The conserved domain of BBX proteins was identified using the NCBI CDD tool (https://www.ncbi.nlm.nih.gov/Structure/cdd/, accessed on 20 August 2023). Phylogenetic trees, conserved motifs, conserved domains, and gene structures of 125 BBX proteins were analyzed and visualized using Gene Structure View (Advanced) of TBtools v2.096 software [51].

### 4.4. Chromosomal Localization and Collinearity analysis of BBX

Based on the three genome annotation data of *M. sativa* spp. *Caerulea*, *M*. *truncatula*, and *M*. *sativa*, the chromosome locations of BBXs were identified and mapped using the Gene Location visualization of TBtools software. Based on the gene location, the detected genes were named *MaBBX1*~*MaBBX21*, *MtBBX1*~*MtBBX25*, and *MsBBX1*~*MsBBX79*, respectively. The downloaded genome sequence files and GFF files were analyzed using One Step MCScanX—super Fast of TBtools software to obtain the genome sequence files of the three genomes of *M*. *sativa* spp. *Caerulea*, *M*. *truncatula*, and *M*. *sativa* within the *BBX* gene collinearity information. All output files were then imported into TBtools v2.096 software of TBtools to obtain visualization of the collinearity relationships among *BBX* family members. The homologous genes of these three *Medicago* species were analyzed separately using TBtools software One Step MCScanX—super Fast. The Simple Ka/Ks Calculator (NG) of TBtools software was used to calculate the non-synonymous substitution rate (Ka) and synonymous substitution rate (Ks) of *BBX* gene pairs [51].

### 4.5. Analysis of Expression Level of MsBBX Gene

The original transcriptome data of six different tissues of *M. sativa* were obtained in a previous study under NCBI (https://www.ncbi.nlm.nih, accessed on 17 September 2023) project ID PRJNA276155. The original transcriptome data were also obtained for cold treatment, cold and salicylic acid treatment, salt treatment, drought treatment, heat treatment, abscisic acid treatment, and salt and jasmonic acid treatment in projects PRJNA450305, PRJNA573724, PRJNA867517, PRJNA1019078, and PRJNA1070376, respectively.

The data were then converted into fastq files with SRA-Toolkit v2.9 (NCBI, USA). Raw reads were trimmed using Trimmomatic-0.39 [52]. The gene expression level was determined by mapping cleaned reads to the corresponding *M. sativa* reference genomes using the StringTie v2.1.3 package (GitHub, San Francisco, USA) [53].

TPM (transcripts per million) indicates the gene expression level [54]. Heat maps were produced using TBtools software and differential expression of *MsBBXs* was analyzed using the DESeq2 R package. Genes with an adjusted *p*-value < 0.01 and |log2 foldchange (FC)| > 1 according to DESeq2 were designated as differentially expressed genes.

### 4.6. Plant Materials

The XinJiangDaYe variety of alfalfa was used in this study; the seeds were obtained from Dr. Yanhui Zhang of the College of Prataculture and Environmental Sciences, Xinjiang Agricultural University (Urumqi, China). Seedlings were planted in humus soil and grown in a greenhouse with a 16/8 h day/night photoperiod at 25 °C. Alfalfa seedlings of the same size and growth at three months of age were selected as treatment material and continued in Hoagland’s solution for 14 days. Five treatments were set up: 150 mmol/L of NaCl, 0.5 mmol/L of SA, 0.5 mmol/L of ETH, 150 mmol/L of NaCl and 0.5 mmol/L of SA, and 150 mmol/L of NaCl and of 0.5 mmol/L ETH. Three biological replicates were set up for each treatment, with 15 alfalfa seedlings per replicate. At five treatment periods of 0 h, 3 h, 6 h, 12 h, and 24 h, root materials from each treatment group were collected and immediately placed in liquid nitrogen, then stored at −80 °C for subsequent experimental analyses.

### 4.7. Total RNA Extraction and qRT-PCR Analysis

Quantitative real-time PCR (qRT-PCR) was performed using TRIzol reagent (Invitrogen, Carlsbad, CA, USA) to extract total RNA from the samples. cDNA was synthesized using the FastQuant First Strand cDNA Synthesis Kit (Tiangen, Beijing, China) according to the manufacturer’s protocol. The qRT-PCR was performed using a LightCycler 480 Real-Time PCR system (Roche, Basel, Switzerland), an SYBR^®^ Green Premix Pro Taq HS qPCR kit(Accurate Biotechnology, Hunan, China, and a Roche LightCycler instrument. Each treatment had 3 biological replicates. The qRT-PCR primers were designed using Premier5 software v5.00 (PREMIER Biosoft, San Francisco, USA) (Table S4). MsEF-1α was used as an internal reference gene. The 2^−∆∆Ct^ method was used to compute the relative expression. Statistical analysis was performed using SPSS 22.0 software (SPSS Inc., Chicago, IL, USA). Statistical differences between measurements at different times or with different treatments were analyzed using Duncan’s multiple range test. Differences were considered significant at a probability level of *p* < 0.05.

### 4.8. Subcellular Localization

*Nicotiana benthamiana* seeds were obtained from the Key Laboratory of Xinjiang Phytomedicine Resource and Utilization of Ministry of Education in Shihezi University (Shihezi, China). To investigate the transient expression of *MsBBXs* in tobacco leaves, the full-length CDS removes the terminator. *MsBBXs* were PCR-amplified using primers containing kpn I and xba I restriction endonucleases (see Supplemental Table for the sequences) and ligated into the vector pCAMBIA1300-eGFP cleaved by kpn I and xba I enzymes. The constructed vector was transformed into *Agrobacterium rhizogenes* GV3101, infiltrated into 4-week-old *Nicotiana benthamiana* leaves, and dark-incubated for 48 h. The fluorescence signals of the epidermis of *Nicotiana benthamiana* leaves were observed using a confocal microscope (Nikon, Japan). *Agrobacterium* strains carrying recombinant plasmids were grown in a liquid LB medium. The experiments used pCAMBIA1300-35S-mCherry-NLS (Puint, Shaanxi, China) as a marker for the nucleus. 

## 5. Conclusions

In our study, 125 *BBX* family members were identified in the genomes of three *Medicago* plants: 21 *MaBBXs*, 25 *MtBBXs*, and 79 *MsBBXs*. Their basic structures, conserved motifs, protein physicochemical properties, and chromosomal localization information were analyzed. According to the phylogenetic relationship, these *BBXs* are divided into five evolutionary branches, with the largest number of *BBXs* in Clade Ⅰ, with 44 members. WGD or segmental duplications and dispersed duplications were the main ways of family member duplication; 5, 5, and 106 pairs of homogenic genes were calculated in the *MaBBX*, *MtBBX,* and *MsBBX* genomes, respectively. Except for two gene pairs of Ka/Ks > 1, which experienced positive selection, the rest of the collinear gene pairs had Ka/Ks < 1, suggesting that most of the *BBXs* experienced strong purifying selection. We also analyzed the expression patterns of *BBX* genes in different tissues as well as under multiple abiotic stresses. Some *BBX* genes significantly induced by salt and hormones were detected by qRT-PCR. Subcellular localization experiments revealed that *BBX* genes may act as transcription factors to regulate the transcription of genes in the nucleus. These results provide a theoretical basis for insight into the evolution and function of *BBX* family genes in alfalfa. It lays a theoretical foundation for the study of the resistance performance of *Medicago* plants.

## Data Availability

Data are contained within the article and supplementary materials.

## Supplementary Materials

The supporting information can be downloaded at https://www.mdpi.com/article/10.3390/ijms25115778/s1.
