# Supplementary material for: Genome-Wide Analysis of BBX Gene Family in Three Medicago Species Provides Insights into Expression Patterns under Hormonal and Salt Stresses
Source: Int J Mol Sci. 2024 May 26;25(11):5778. doi: 10.3390/ijms25115778 (PMC11171683; doi:10.3390/ijms25115778)
Supplement: Supplementary file 1 [file ijms-25-05778-s001.zip › Supplementary Materials/Figure S1 Gene Structure, Conserved Domains and Motif Composition of BBXs Genes.pdf]

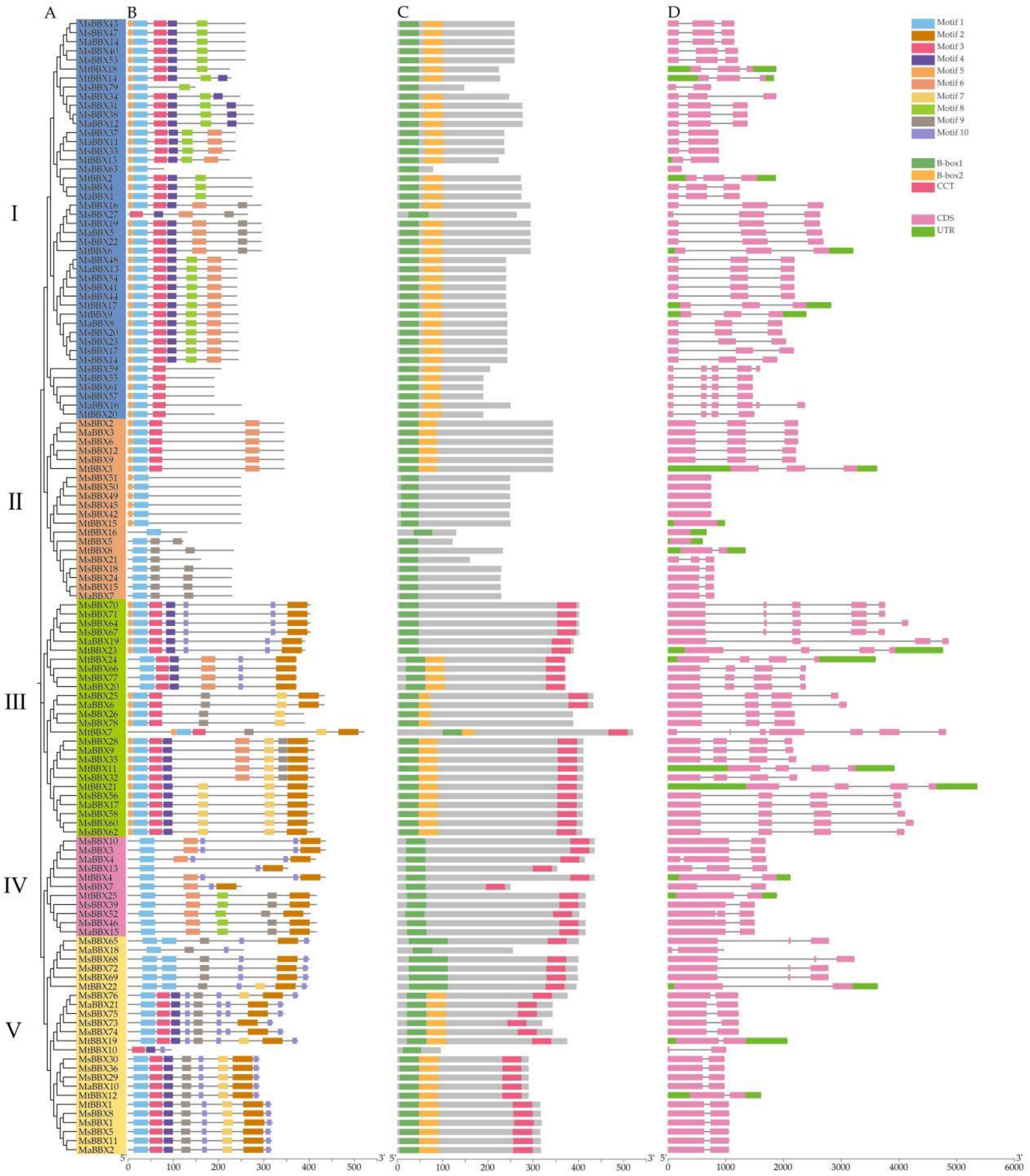

Figure S1. Gene Structure, Conserved Domains and Motif Composition of *BBXs* Genes. (A) The phylogenetic tree was built by the NJ method with a bootstrap value of 1000. (B) The conserved motifs in BBX proteins (1-10) are in different colors. The black lines represent relative protein lengths. (C)The conserved domain of BBX proteins. The green, yellow and pink boxes represent the B-box 1, B-box 2 and CCT domains, respectively. (D) Exons, introns and untranslated regions (UTR) are represented by pink rectangles, gray lines and green rectangles, respectively.
